# Pharmacological evidences for the blood pressure lowering and cardiovascular inhibitory actions of the essential oil of *Thymus serrulatus* hochst. Ex benth

**DOI:** 10.3389/fphar.2025.1719712

**Published:** 2026-01-08

**Authors:** Najeeb Ur Rehman, Mohd Nazam Ansari, Abdulrahman A. Aldossari, Thamer A. Alhatlan, Amber Hanif Palla, Aman Karim, Muhammad Noman

**Affiliations:** 1 Department of Pharmacology and Toxicology, College of Pharmacy, Prince Sattam Bin Abdulaziz University, Al-Kharj, Saudi Arabia; 2 Department of Biological and Biomedical Sciences, The Aga Khan University Hospital and Medical College, Karachi, Pakistan; 3 Department of Biological Sciences, National University of Medical Sciences Rawalpindi, Rawalpindi, Pakistan; 4 Department of Pharmacy, Quaid-i‐ Azam University, Islamabad, Pakistan

**Keywords:** alpha-1 receptor antagonist, aorta, atria, hypertension, *T. serrulatus*, vasodilator

## Abstract

**Background:**

*T. serrulatus* is used in folk medicine for the treatment of cardiovascular disorders, including hypertension. This study investigates its hypotensive, cardiac-depressant, and vasodilatory activities.

**Methods:**

The hypotensive effect of *Thymus serrulatus* essential oil was evaluated *in vivo* in anesthetized rats by measuring changes in mean arterial blood pressure following intravenous administration. *Ex vivo*, cardiac-depressant activity was assessed in isolated guinea-pig atrial preparations, and vasodilatory effects were examined in rat aortic rings.

**Results:**

Intravenous administration of *T. serrulatus* essential oil produced a dose-dependent (1–10 mg/kg) reduction in arterial blood pressure. In spontaneously beating guinea-pig atrial tissues, the oil exerted negative chronotropic and inotropic effects at concentrations of 0.1–5 mg/mL. In rat aorta, it caused complete relaxation of phenylephrine (PE, 1 μM)-induced contractions, with an EC_50_ of 1.27 mg/mL, while partial relaxation (59% ± 3%) was observed against high K^+^ (80 mM). The vasodilatory effect against PE was not significantly altered by endothelium removal or atropine pretreatment, indicating an endothelium- and muscarinic-independent mechanism. Preincubation with a lower concentration (0.1 mg/mL) produced a rightward shift in PE-mediated concentration–response curves (CRCs) without reducing maximal response, similar to prazosin-like competitive antagonism. A higher concentration (0.3 mg/mL) suppressed the maximal PE response, consistent with non-competitive antagonism comparable to verapamil. In Ca^++^-free medium, preincubation with *T. serrulatus* (0.3 and 1 mg/mL) shifted Ca^2+^ CRCs to the right with reduced maximal response, further supporting Ca^++^ channel–blocking activity.

**Conclusion:**

*T. serrulatus* essential oil exhibits hypotensive, cardiac-depressant, and vasodilatory effects, likely mediated through α-adrenergic antagonism and Ca^++^ channel blockade. These findings provide pharmacological support for its traditional use in cardiovascular disorders, including hypertension.

## 1 Introduction

Hypertension continues to be a leading cause of premature mortality globally affecting an estimated 1.4 billion adults aged 30–79 years (33%) worldwide. Despite its burden, about 44% of them are unaware of their condition, and the ones who receive treatment, only 320 million (23%) have their blood pressure adequately controlled (WHO Key facts, 2024; World Health Organization, 2025). Nearly, two-thirds of those affected live in low- and middle-income countries.

Uncontrolled hypertension not only contributes to heart attacks, heart failure, strokes, chronic kidney disease, dementia, and other serious complications but also results in substantial socioeconomic costs (Ringwald-de Meyer et al., 2025; Fa et al., 2025). Conventional pharmacological therapies, while effective, often require lifelong adherence to multiple costly medications, creating barriers to accessibility for low-income populations and increasing the risk of adverse effects such as electrolyte imbalances and renal dysfunction (Mills et al., 2016; Deepshikha et al., 2025).

Medicinal plant derived natural products serve as a promising adjunct or alternative therapeutic approach, leveraging their ability to target interconnected pathological pathways such as oxidative stress and endothelial dysfunction through multimodal mechanisms while maintaining a relatively favourable safety profile (Adaramoye et al., 2009). This approach aligns with contemporary hypertension management guidelines, such as the 2021 WHO recommendations, which emphasize lifestyle modifications including dietary interventions rich in plant-based bioactive metabolite(s),as first-line interventions for stage 1 hypertension or as complementary therapy for higher-risk patients (WHO World Health Organization, 2021). European Society of Cardiology (Del Pinto et al., 2023) also recognize non-pharmacological strategies to be used for individuals with prehypertension or low-to-moderate cardiovascular risk, to reduce reliance on polypharmacy and improve long-term outcomes.

Functional foods, which provide health benefits beyond basic nutrition, are a key dietary strategy for disease management. Medicinal plants of the genus thymus has traditionally been used worldwide for various ailments including cardiovascular disorders (Anwar et al., 2024; El Hachlafi et al., 2021). The scientific studies on the extracts and isolated metabolites from various species of thymus indicates their antihypertensive and cardioprotective effects (Mihailovic-Stanojevic et al., 2013; Auger et al., 2018; Patil et al., 2021; Geleta et al., 2015). One example is *T. serrulatus (Thymus serrulatus;* locally known as Tesin), a plant native to Ethiopia that has been traditionally used to treat cardiovascular conditions (Melka et al., 2016) and their role as a food additive, in cuisines (Damtie and Mekonnen, 2015; Meresa et al., 2017).

Previous studies on *T. serrulatus* have primarily evaluated its aqueous leaf extract, which demonstrated dose-dependent vasodilation in isolated aortic tissues of rats. This effect was attributed to a K^+^-channel opening (KCO)-like mechanism (Geleta et al., 2015), suggesting a possible role in reducing peripheral vascular resistance (PVR). However, any intervention that lowers PVR alone may trigger reflex tachycardia, thereby limiting clinical usefulness. Therefore, it is essential to investigate these mechanisms *in vivo*, where integrated cardiovascular reflexes operate. Because blood pressure (BP) is determined by both cardiac output (CO) and PVR, clarification of whether the antihypertensive effect of *T. serratulus* arises from (i) reduced cardiac contractility or heart rate (as seen with agents like verapamil or propranolol), (ii) vascular relaxation through smooth muscle (as with amlodipine or prazosin), or (iii) a combination of both pathways (as with diltiazem), is necessary to define its pharmacological profile.

Additionally, the aqueous extract alone does not fully represent the pharmacological potential of the plant, as *T. serrulatus* contains a diverse mixture of polar and non-polar metabolites. It is well established that plant extracts often exhibit differential biological activities across polarity fractions with polar extracts frequently showing stronger smooth muscles stimulation and antioxidant effects, whereas non-polar (lipophilic) fractions demonstrate more pronounced inhibitory actions on smooth muscles, antibacterial and/or membrane-active properties (Khan et al., 2012; Gilani et al., 2010; Chaudhary et al., 2012; Rayan et al., 2020). Therefore, limiting investigations to only the aqueous (polar) extract provides an incomplete understanding of the plant’s therapeutic potential. To elucidate the true spectrum of cardiovascular effects, both polar and non-polar fractions, particularly the essential oil, require systematic evaluation.

In our earlier work, GC–MS analysis of *T. serrulatus* essential oil identified several bioactive metabolites (Rehman et al., 2021), including carvacrol, thymol, and (−)-borneol. These metabolites have been independently reported to exhibit antihypertensive, vasorelaxant, and cardio-suppressant activities in various experimental models (Khazdair et al., 2024; Barreto da Silva et al., 2020; Peixoto‐Neves et al., 2010; Silva‐Filho et al., 2012). Despite this promising phytochemical profile and detailed evaluation of the antihypertensive activity of its aqueous extract, the essential oil of *T. serrulatus* itself has not been systematically investigated for its cardiovascular actions or underlying mechanisms. Therefore, the present study aimed to evaluate the pharmacological effects of *T. serrulatus* essential oil on vascular and cardiac tissues and to elucidate the detailed mechanism(s) by which it may exert antihypertensive activity, thereby providing a mechanistic basis for its traditional use in hypertension.

## 2 Materials and methods

The study was designed in a step-wise manner to systematically evaluate the cardiovascular actions of *T. serrulatus* essential oil. The plant material was first collected, authenticated, and hydro-distilled to obtain a standardized essential oil suitable for pharmacological testing. *In vivo* blood pressure studies in anesthetized rats were performed first, as these provided the primary evidence of a hypotensive effect and established physiologically relevant doses. Based on the observed fall in arterial pressure, *ex vivo* cardiac studies were then conducted in isolated guinea-pig atria to determine whether the hypotensive response involved cardiac depressant actions (negative chronotropic/inotropic effects). Finally, mechanistic vascular studies were carried out using rat aortic rings to investigate whether vasodilation contributed to the antihypertensive effect and to differentiate between receptor-mediated and calcium channel–mediated pathways. This sequential workflow ensured a logical progression from whole-animal responses to isolated-tissue mechanisms.

### 2.1 Plant material and extraction

The extract was prepared from the fresh aerial parts of *T. serrulatus* which were collected during October 2018, which is considered as the flowering times. These plants were collected from the Amba Alaje mountain area in South Tigray, Ethiopia (12° 59′54.4″N, 39° 32′52.3″E). The identification was confirmed using the Flora of Tropical Africa (Patil et al., 2021). They were further authenticated by the Botanist, Dr Getinet Masresha from the Department of Biology, University of Gondar. The specimen has been deposited at the herbarium of the University of Gondar with voucher number TH-001/2011.

The essential oil was obtained by hydro-distillation of finely cut pieces of the herb for 3 h by using a Clevenger-type apparatus eleven times. Anhydrous sodium sulfate was used to dry the oil and the final yield was 0.9% v/w (24.75 mL) of essential oil. The oil was then stored in tightly sealed vials at 4 °C (Asfaw et al., 2000). Before the assays, the oil was dissolved in Tween 80 in saline/Krebs (1% w/v) and sonicated as per the requirement of the assay.

### 2.2 Experimental animals

Adult Sprague-Dawley rats (180–200 g) and local breed guinea pigs weighing between 450 and 500 g and of either sex were used. The animals were maintained in the standard conditions of light and dark cycle (23 °C–25 °C), with a standard diet and water supply (*ad libitum)*. The animal studies were designed by ensuring the care of animals in line with not only the national and international guidelines but also the institutional guidelines including approval from the Standing Committee of Bioethics Research (SCBR) at Prince Sattam Bin Abdulaziz University (reference number SCBR-394/2024). Rats and guinea pigs were sacrificed by isoflurane overdose. All protocols complied with ARRIVE guidelines for animal use.

### 2.3 Chemicals

The isolated organ bath chemicals were purchased from Sigma Chemical Company, St. Louis, MO, United States) and included acetylcholine chloride (Ach), verapamil, isoprenaline hydrochloride, norepinephrine (NE), phenylephrine hydrochloride (PE), and prazosin. Pentothal sodium (thiopental) and isoflurane were obtained from Abbot Laboratories, Karachi, Pakistan. All the drugs were made up fresh in distilled water as concentrated stock solutions so that the total volumes added to the 20 mL organ baths never exceeded 0.65 mL.

### 2.4 Measurement of BP in anesthetized rats

Rats were anesthetized (Gilani et al., 2005) with thiopental sodium (70–90 mg/kg; IP) followed by tracheal cannulation using a polyethylene (Pe)-20 tubing. The right jugular vein was cannulated with PE-50 tubing for intravenous administration of drugs, and the left carotid artery was cannulated and connected to a reusable pressure transducer (MLT 0380/D Reusable BP-Transducer) filled with heparinized saline (60 IU/mL) to prevent clottingThe arterial pressure was amplified using a Quad Bridge Amplifier and digitalized by Power-Lab ML 4/25 data acquisition system (AD Instruments, Sydney, Australia). The cannulated area was kept moist using saline-soaked guaz. Before injecting drugs/test substances into the jugular vein, 0.1 mL of heparin-treated saline was flushed through the IV tubing to dissolve any blood clots and ensure smooth drug delivery. After setting up all equipment and cannulas, the preparation was allowed to stabilize for at least 20 min to ensure a steady heart rate. Once stable, the test substance was injected intravenously, and blood pressure (BP) was monitored. Before giving the next dose, BP was allowed to return to its baseline level. A substance was classified as antihypertensive if it consistently reduced blood pressure compared to pre-injection values. The mean arterial pressure (MAP) was calculated as:

MAP = Diastolic BP + 1/3 pulse pressure.

Pulse pressure = Systolic BP - diastolic BP.

If the lowest recorded value after administration differed measurably from the baseline (pre-injection) resting value, it was considered to alter mean arterial pressure (MAP).

### 2.5 Effect on isolated Guinea-pig atria

To evaluate the cardiac inhibitory activity of the test substance, guinea-pigs (450–500 g) preferably male were sacrificed by cervical dislocation and their right atria were isolated and studied using established methods (Shah et al., 2024). Each atrium was placed in 20 mL tissue baths containing Krebs solution (aerated with carbogen gas and maintained at 32 °C). Tissue preparation, from animal sacrifice to atrium placement in the warmed Krebs solution, took approximately 45 min. Guinea pig right atria retain spontaneous rhythmic contractions due to intrinsic pacemaker activity (Taqvi et al., 2006). Before testing, the atrium was stabilized for 45 min under 1 g resting tension. Isometric contractions were recorded using a force sensor connected to a Power Lab system and computer. The test substance was deemed effective if it reduced both the atrial contraction rate and force compared to baseline values, with results expressed as percentage changes from baseline.

### 2.6 Effect on rat aorta preparations

Previously defined protocols were followed to evaluate the effect of test material on rat aortic rings (Tep-Areenan and Sawasdee, 2011). Rats were euthanized by an overdose of isoflurane, after which the thoracic aorta was excised, cleaned off connective tissues and cut into 5 mm rings that were mounted individually in a 5 mL tissue bath pre-filled with Kreb’s solution.

Tension was monitored using an isometric force transducer (Fort-10, WPI) amplified with a Transbridge TBM 4M amplifier, and digitalized through a PowerLab ML 845 system. Rings were stabilized at 1 g resting tension for 60 min, with Krebs solution refreshed every 15 min to prevent metabolite accumulation (Altura and Altura, 1970).

Endothelial integrity was assessed with acetylcholine (10 µM), where >90% relaxation indicated intact endothelium and <10% indicated denudation.

Mechanistic evaluation involved testing the material against phenylephrine (PE, 1 µM) and high K^+^ (80 mM)-induced contractions. Inhibition of PE-induced contraction indicated receptor-operated Ca^2+^ channel blockade, whereas inhibition of high K^+^ contraction suggested L-type Ca^2+^ channel blocking (CCB) activity.

### 2.7 Statistical analysis

Data analysis, graphing, and calculations were performed using GraphPad Prism 4.00 (GraphPad Software, San Diego, CA, United States). Results are expressed as mean ± standard error of the mean (SEM). Median EC_50_ values were derived using geometric means with 95% confidence intervals (CI). Concentration-response curves (CRCs) were analyzed via nonlinear regression. Statistical significance was defined as *p* < 0.05.

## 3 Results

### 3.1 Antihypertensive effect of *T. serrulatus*

**FIGURE 1:**
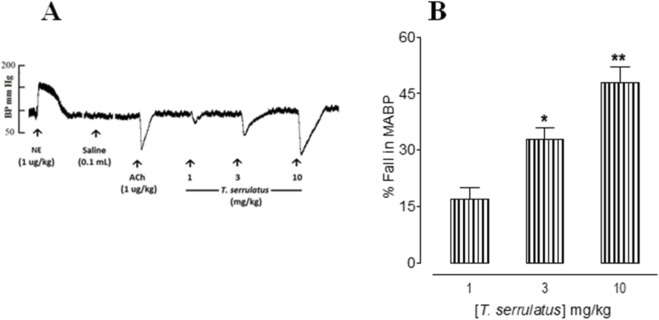
Panel **(A)** shows a typical tracing of the essential oil extracted from *Thymus serrulatus* (*T. serrulatus*) blood pressure (BP)-lowering effect and Panel **(B)** shows a bar chart representing the hypotensive effect of *T. serrulatus* on mean arterial blood pressure (MAP) in anesthetized rats. The dose was administered after the response to the preceding one had returned to normal. Values shown represent mean ± SEM, n = 6.

The essential oil of *T. serrulatus* produced a clear, dose-dependent hypotensive effect in anesthetized rats. Increasing doses (1–10 mg/kg) reduced MAP by 17% ± 3%, 33% ± 3%, and 48% ± 4%, corresponding to absolute decreases of 17 ± 3 mmHg, 33 ± 3 mmHg, and 48 ± 4 mmHg, respectively. A representative arterial pressure tracing illustrates the acute fall in MAP following administration of the oil (Figure 1A), while pooled data confirm a consistent dose-dependent reductions across experimental replicates (Figure 1B).

### 3.2 Effect on rate and force of contraction

**FIGURE 2:**
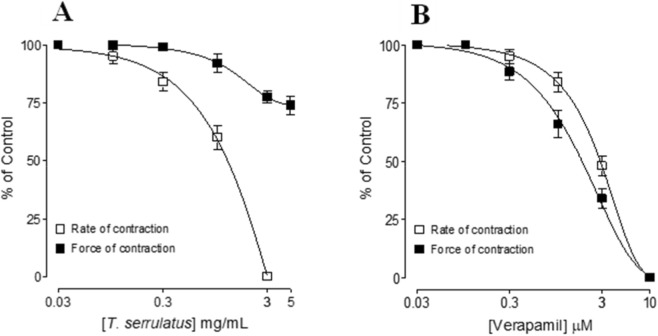
Inhibitory effect of the essential oil extracted from *Thymus serrulatus* (*T. serrulatus*) **(A)** and verapamil **(B)** on force and rate of spontaneous contractions in isolated guinea pig right atria. The values shown are mean ± SEM, n = 6-7.

In isolated guinea-pig right atria, *T. serrulatus* essential oil caused a concentration-dependent suppression of spontaneous rate of atrial contraction rate with an EC_50_ of 2.32 mg/mL (95% CI: 1.84–3.62; *n* = 6–7). The contractile force was decreased by 26% ± 4% at highest tested dose of 5 mg/mL. Verapamil, tested in parallel as a reference Ca^+2^ channel blocker, inhibited both force and rate with greater potency (Figures 2A,B). These findings suggest a negative chronotropic effect with less effect on ionotropic effect. This effect indicates possible cholinergic receptor mediated activation, preceding cardio suppressant effect.

### 3.3 Vasodilator effect on rat aorta

**FIGURE 3:**
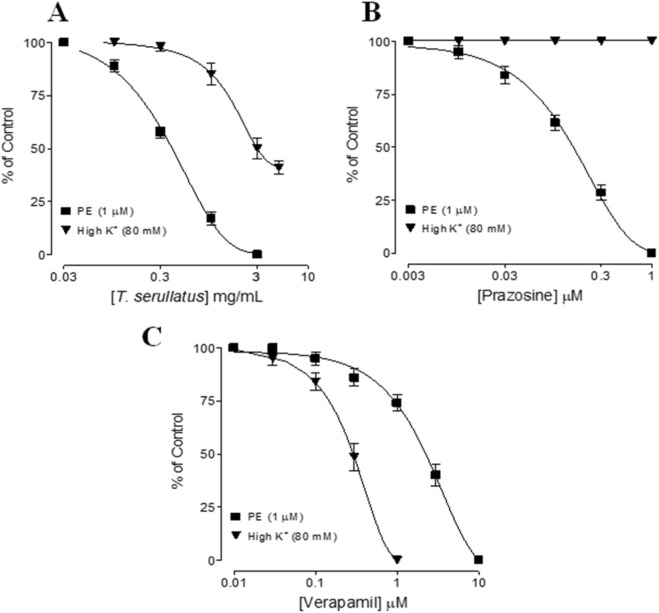
Concentration-dependent relaxant effects of **(A)** the essential oil extracted from *Thymus serrulatus* (*T. serrulatus*), **(B)** prazosin, and **(C)** verapamil on phenylephrine (PE) and high K^+^-induced contractions in isolated rat aortic ring preparations. Values shown are mean ± SEM, n = 5-6.

The essential oil exhibited no intrinsic contractile activity but produced robust vasorelaxation in PE-contracted rat aortic rings. It fully inhibited PE-induced contractions with an EC_50_ of 1.27 mg/mL (95% CI: 0.96–1.82; n = 5–6), whereas relaxation of high K^+^ (80 mM)-induced contractions was partial (59% ± 3% at 5 mg/mL; *n* = 5–6) suggesting preferential inhibition of receptor operated Ca^+2^ entry (Figure 3A). The potency profile resembled that of prazosin against PE and contrasted with verapamil, which more effectively inhibited high-K^+^ contractions (Figures 3B,C).

**FIGURE 4:**
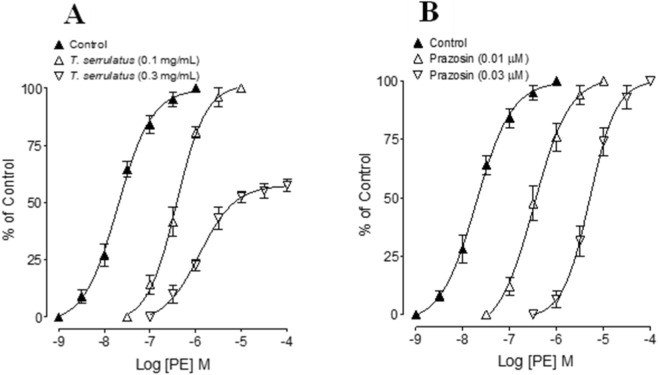
Concentration-response curves of phenylephrine (PE) in the absence and presence of different concentrations of **(A)** the essential oil extracted from *Thymus serrulatus* (*T. serrulatus*) and **(B)** prazosin in isolated rat aortic ring preparations. Values shown are mean ± SEM, n = 5-6.

Pre-incubation with *T. serrulatus* oil (0.1–0.3 mg/mL) produced a concentration-dependent parallel rightward shift of the PE concentration-response curves (CRCs) without reducing maximal contraction, similar prazosin, confirming competitive α1-adrenoceptor antagonism. At the highest concentration (0.3 mg/mL), a reduction in maximal response (57.5% ± 2.5%) also became evident, indicating an additional non-competitive component (Figures 4A,B).

**FIGURE 5:**
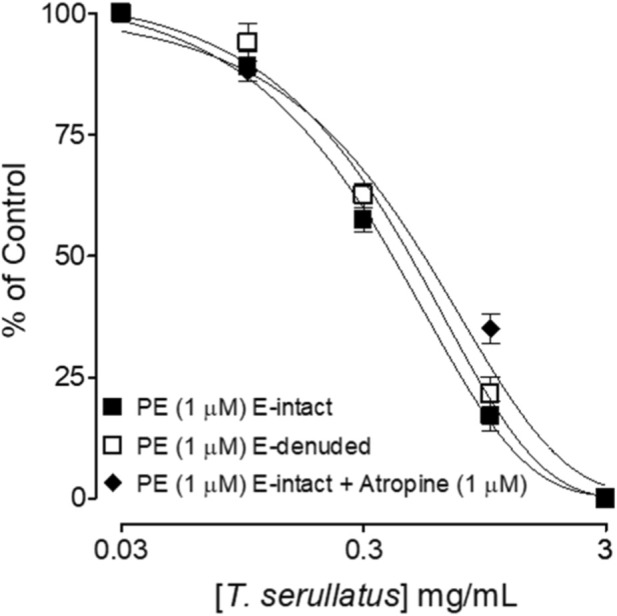
Concentration-dependent relaxant effects of the essential oil extracted from *Thymus serrulatus* (*T. serrulatus*) on phenylephrine (PE)-induced contractions in the absence and presence of atropine (1 µM) in the endothelium (E)-intact and E-denuded isolated rat aortic ring preparations. Values shown are mean ± SEM, n = 6.

Endothelium-intact and endothelium-denuded tissues showed no significant difference in EC_50_ values, and atropine did not modify responses, indicating that vasorelaxation is independent of endothelial mediators or cholinergic pathways (Figure 5).

**FIGURE 6:**
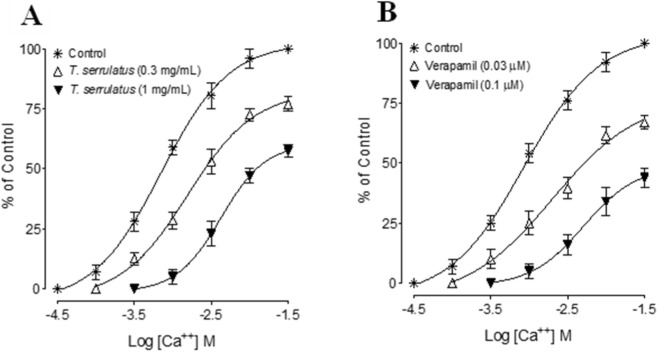
Concentration-response curves of Ca^++^ in the absence and presence of the increasing concentrations of **(A)** essential oil extracted from *Thymus serrulatus* (*T. serrulatus*) and **(B)** verapamil in isolated rat aortic ring preparations. Values shown are mean ± SEM, n = 6.

Finally, the oil produced a non-parallel rightward shift with depression of maximal responses in Ca^2+^ CRCs (0.3–1 mg/mL), resembling verapamil (0.03–1 µM), and demonstrating non-competitive blockade of voltage-dependent Ca^2+^ channels (Figures 6A,B).

## 4 Discussion

The present study provides new pharmacological evidence for the cardiovascular actions of *T. serrulatus* and helps clarify mechanisms that have remained insufficiently defined despite its long-standing traditional use in hypertension. (Demissew, 1993; Parvenz and Yadav, 2010). Although Geleta et al. (Geleta et al., 2015) previously demonstrated antihypertensive effects of *T. serrulatus* using aqueous extracts, the mechanistic basis of these observations remained largely speculative, in part because aqueous preparations capture only the polar metabolites of the botanical drug. In contrast, the essential oil is enriched in lipophilic bioactive molecules that are not extracted in water-based preparations. By evaluating the essential oil, our study provides a more complete pharmacological characterization of the botanical drug’s antihypertensive potential and elucidates the mechanistic pathways underlying its vasodilatory and cardio-depressant effects.

*T. serrulatus* essential oil reduced the rate and force of contraction of heart in anesthetized rats with reduction in MAP. To evaluate whether the effect of the oil is mediated by reducing PVR, the effect against PE-induced contraction in the rat aorta was recorded. PE is a known alpha-adrenergic agonist and aorta which contains alpha-1 receptors (Pérez-Vizcaíno et al., 1998). Alpha-1 receptors are the G-protein coupled receptors, and their activation causes an increase in intracellular calcium leading to vasoconstriction. We found that increasing doses of the essential oil completely reversed the PE-mediated contraction, indicating the mechanism to be mediated by alpha-1 blockade. To confirm whether this antagonism was due to competitive or non-competitive inhibition, CRCs were plotted for PE in the presence and absence of the low dose (0.1 mg/mL) of essential oil. There was a rightward parallel shift, similar to prazosin, a competitive α-1 antagonist (Doggrell, 1992). Since this effect is mediated at the lower dose, it indicates the main antihypertensive mechanism of *T. serrulatus* may be by reducing the PVR. At a higher dose (0.3 mg/mL), a downward non-parallel shift was observed indicating the presence of an additional mechanism. These findings align with our prior reports demonstrating the CCB effect on the tracheal tissues (Rehman et al., 2021). Although the CCB-like activity of this botanical drug was less pronounced in rat aorta it completely relaxed high K^+^-induced contractions in guinea pig trachea, suggesting species- or tissue-dependent variability (Xiao et al., 2006; Rehman et al., 2011). The differences in calcium channel subtypes in different tissues could be one of the reasons as vascular tissue is rich in T-type channels (Katz, 1996), while cardiac tissues are rich in L-type channels—more sensitive to verapamil. Thus, the oil’s cardio-depressant effects were due to its predominant effect on L-type Ca^+2^ channels.

Vasodilation is also mediated through endothelium-dependent factors. Hence, we also tested the extract for its role in those mechanisms. We found no difference in vasodilatory effect in the intact and denuded preparations of rat aorta, which suggests that the blood pressure-lowering effect of *T. serrulatus* oil is independent of the L-arginine-NO pathway (Palla et al., 2021). Our finding contrasts with previous reports by Geleta et al. (Geleta et al., 2015) who demonstrated that the aqueous extract of *T. serrulatus* induced endothelium-dependent vasodilation in guinea pig aorta in a concentration-dependent manner. One of the reasons for the difference in effect is the difference in the type of formulations as the later used aqueous extracts which have a divergent phytochemical profile as compared to essential oil. Secondly, Gelata et al. (Geleta et al., 2015) reported this effect on guinea pigs’ aorta whereas we evaluated these effects on rat aorta. We have previously shown species-specific variations (rat vs. guinea pig) (Gilani et al., 2013; Rehman et al., 2012) based on which the current findings imply that *T. serrulatus* essential oil may exhibit tissue-selective mechanisms. In our study, *T. serrulatus* essential oil relaxes PE- and high K^+^-induced contractions, indicating an inhibitory effect on voltage-dependent Ca^+2^ entries. However, because isolated vascular preparations (rat aorta) express predominantly T-type Ca^2+^ channels, whereas cardiac tissues of guinea pig atria are enriched in L-type Ca^2+^ channels, the distinction between channel subtypes in our study is primarily inferred from tissue selectivity rather than directly confirmed through subtype-specific antagonists or electrophysiological assays. The robust relaxation of PE-induced contraction at low concentrations suggests α1-antagonism as the primary vascular mechanism, while the negative inotropic and chronotropic effects in atrial tissue imply additional inhibition of L-type channels. Taken together, these findings suggest a concentration-dependent transition rather than additive synergy between α1-adrenergic blockade and Ca^+2^ channel inhibitions. At lower doses, α1 antagonism predominates, producing vasodilation and reduction in peripheral resistance, whereas at higher doses, concurrent L-type Ca^+2^ channel blockade contributes a negative inotropic and chronotropic component that further lowers mean arterial pressure. Physiologically, this sequential recruitment of vascular and cardiac mechanisms may underlie the graded antihypertensive response observed *in vivo*, reflecting a dose-dependent pharmacodynamic continuum rather than distinct additive effects. One of the adverse effects of reducing PVR is reflex tachycardia, hence to ensure that *T. serrulatus* essential oil does not cause this adverse effect we also evaluated the effect in isolated guinea pig atria, as this tissue has self-rhythmic potential. We found that the oil decreased heart rate partially and suppressed contraction force completely in isolated guinea-pig atria. Hence, taken together as a whole, and based on the findings of this study, we are reporting for the first time that *T. serrulatus* essential oil mediates its antihypertensive and vasodilatory effect predominantly mediated via the alpha-1 antagonism and blockade of T-type Ca^+2^ channels. It also affects cardiac output by reducing the rate and force of contraction thus counteracting the reflex tachycardia.

Findings from other *Thymus* species also support the cardiovascular actions demonstrated in the present study. For example, *Thymus vulgaris* aqueous extract has been shown to exert antihypertensive and vasculoprotective effects in renovascular hypertensive rats, where chronic supplementation lowered systolic blood pressure and ameliorated hypertension-associated biochemical disturbances such as elevated creatinine and cholesterol levels (Kensara et al., 2013). Importantly, *T. vulgaris* treatment also prevented structural damage to the thoracic aorta, including endothelial irregularities, extracellular matrix expansion, and smooth-muscle hypertrophy, thereby preserving vascular integrity. In addition, recent work on *T. vulgaris* essential oil further supports the antihypertensive potential of *Thymus* species (El-gammal et al., 2025). EL-Gammal et al. (El-gammal et al., 2025) demonstrated that a thymol-rich *T. vulgaris* essential oil significantly lowered blood pressure in hypertensive animal models and provided broad protection against hypertension-induced organ injury, normalization of biochemical parameters, and preservation of vascular and cardiac structure, indicating both functional and structural cardiovascular benefits. Collectively, these findings highlight that both lipophilic and aqueous metabolites of thymol-dominated *Thymus* essential oils are therapeutically important and support the notion contribute substantially to the genus’ antihypertensive and organ-protective effects. The proposed mechanisms are further supported by known structure–activity relationships of thymol and carvacrol, the major *Thymus* monoterpenoids, which have been shown to inhibit Ca^+2^ influx and relax vascular smooth muscle (Boskabady et al., 2011; Silva et al., 2014).

Similarly, *Thymus linearis* extracts have been shown to induce endothelium-independent vasodilation and inhibit agonist-mediated vascular contraction, mechanisms that closely resemble the α1-adrenergic antagonism demonstrated in our study. Notably, while aqueous extracts of *T. vulgaris* and *T. linearis* predominantly highlight NO-mediated or potassium-channel–dependent pathways, our essential oil preparation produced vasodilation that was independent of the L-arginine–NO axis, emphasizing the role of formulation-specific phytochemical differences. Taken together, these comparisons indicate that *T. serrulatus* shares mechanistic themes with other medicinal *Thymus* species but also displays distinct pharmacological features related to its essential oil profile, thereby enhancing its ethnobotanical and therapeutic significance within the genus.

This work represents an essential first-step mechanistic evaluation of *T. serrulatus* essential oil, and further receptor-level, molecular, and *in-vivo* investigations are warranted. Although invasive blood pressure measurement in anesthetized rats provides highly accurate hemodynamic data, the use of anesthesia may blunt autonomic reflexes; therefore, future studies employing conscious telemetry models would improve translational relevance. Likewise, while vasorelaxant and cardio-depressant effects were demonstrated through functional assays, we were unable to perform molecular confirmation including receptor-binding studies, selective channel-blocker assays, or downstream signalling analyses, which limits mechanistic depth. Without selective blockers (e.g., mibefradil for T-type or nifedipine/verapamil for L-type) or patch-clamp confirmation, the involvement of specific channel subtypes remains mechanistic inference. Future studies employing channel-selective pharmacologic tools or ionic current recordings are required to definitively characterize these interactions.

In addition, in the current study, both male and female animals were used without sex-specific subgroup analysis, an important limitation given known sex differences in vascular reactivity and autonomic regulation (Blenck et al., 2016). In addition, the inhibitory effects observed in guinea pig atria could not be fully delineated, and future studies will explore whether β-adrenergic blockade, cholinomimetic activity, or other pathways contribute to the selective suppression of contractile force versus rate. We also did not perform toxicity or safety assessments and selected restricting the translational applicability of the findings. Furthermore, all experiments were performed in healthy animals; antihypertensive responses may differ under conditions such as chronic hypertension, endothelial dysfunction, or altered sympathetic tone. Finally, species- and tissue-specific differences (e.g., rat aorta versus guinea pig trachea and atria) may affect generalizability, and future work should employ a single disease model to comprehensively evaluate BP–regulating mechanisms and tissue selectivity.

**FIGURE 7:**
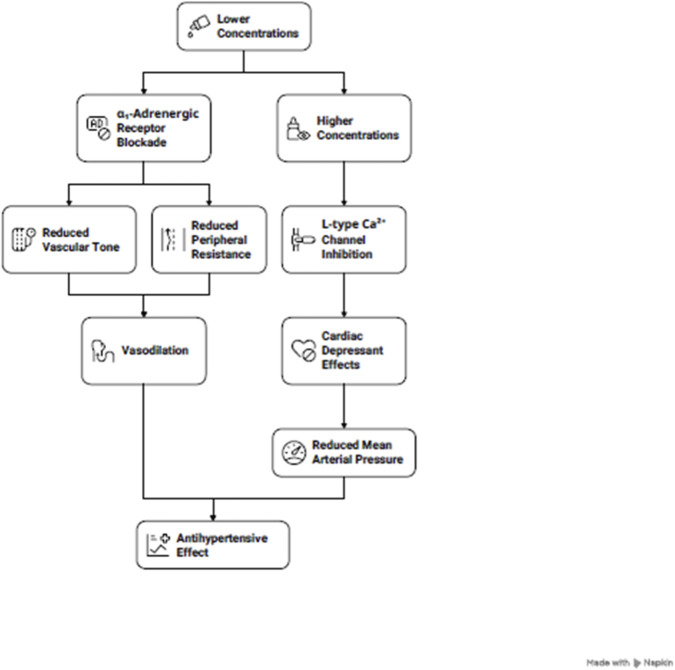
Proposed dose-dependent mechanisms of the essential oil extracted from *Thymus serrulatus* (*T. serrulatus*). At lower concentrations, the oil predominantly blocks α1-adrenergic receptors, reducing vascular tone and peripheral resistance. At higher concentrations, it additionally inhibits L-type Ca^+2^ channels, producing cardiac depressant effects that further reduce mean arterial pressure. Together, these actions constitute a concentration-dependent continuum contributing to the antihypertensive effect.

The proposed mechanism(s) of *T. serrulatus* has been summarized in Figure 7 and Table 1.

**TABLE 1:** Proposed dose-dependent mechanisms of essential oil extracted from *Thymus serrulatus* (*T. serrulatus*).

Concentration range	Primary target	Tissue/Assay model	Mechanism of action	Physiological effect
Low (0.1 mg/mL)	α1-Adrenergic receptors	Rat aortic rings (PE-induced)	Competitive antagonism (rightward shift of PE CRC)	↓ Peripheral vascular resistance (vasodilation)
Moderate (0.3 mg/mL)	α1 + Ca^+2^ channels	Rat aortic rings	Non-competitive antagonism, partial inhibition of Ca^2+^ influx	Enhanced vasodilation
High (≥1 mg/mL)	L-type Ca^+2^ channels	Guinea-pig atria	Negative inotropic and chronotropic effects	↓ Cardiac output, ↓ MAP

## 5 Conclusion

The vasodilatory effect of *T. serrulatus* essential oil is possibly mediated predominantly by competitive inhibition of the alpha-1 adrenergic receptors followed by weak Ca^+2^ channel inhibition and this may also be responsible for the blood pressure lowering effect observed in the *in vivo* studies. Detailed phytochemical characterization, selective chronic *in vivo* models and in-depth molecular *ex vivo* experiments are warranted.

## Data Availability

The raw data supporting the conclusions of this article will be made available by the authors, without undue reservation.
